# Neurophysiological Correlates of Visual Dominance: A Lateralized Readiness Potential Investigation

**DOI:** 10.3389/fpsyg.2017.00303

**Published:** 2017-03-02

**Authors:** You Li, Mingxin Liu, Wei Zhang, Sai Huang, Bao Zhang, Xingzhou Liu, Qi Chen

**Affiliations:** ^1^Center for Studies of Psychological Application and School of Psychology, South China Normal UniversityGuangzhou, China; ^2^Shanghai Kohden Medical Electronic Instrument CorporationShanghai, China; ^3^Epilepsy Center, Guangdong 999 Brain HospitalGuangzhou, China; ^4^Department of Psychology and the Center for Mind and Brain, Guangzhou UniversityGuangzhou, China; ^5^Guangdong Key Laboratory of Mental Health and Cognitive Science, South China Normal UniversityGuangzhou, China

**Keywords:** multisensory competition, visual dominance, the Colavita effect, lateralized readiness potential

## Abstract

When multisensory information concurrently arrives at our receptors, visual information often receives preferential processing and eventually dominates awareness and behavior. Previous research suggested that the visual dominance effect implicated the prioritizing of visual information into the motor system. In order to further reveal the underpinning neurophysiological mechanism of how visual information is prioritized into the motor system when vision dominates audition, the present study examined the time course of a particular motor activation ERP component, the lateralized readiness potential (LRP), during multisensory competition. The onsets of both stimulus-locked LRP (S-LRP) and response-locked LRP (R-LRP) were measured. Results showed that, the R-LRP onset to the auditory target was delayed about 91 ms when it was paired with a simultaneous presented visual target, compared to that when it was presented by itself. For the visual target, however, the R-LRP onset was comparable irrespective of whether it was paired with an auditory target or not. No significant difference was obtained for the onset of S-LRP. Taken together, the time courses of LRPs indicated that visual information was preferentially processed within the motor system, which coincides with the previous finding that the dorsal visual stream prioritizes the flow of visual information into the motor system.

## Introduction

When faced with information from multiple sensory modalities, our brain gives unequal weight to the separate modalities. Usually, the visual modality receives preferential processing and dominates other sensory modalities. One striking example of the dominance of vision over audition is the Colavita effect (Colavita, 1974, 1982; Colavita et al., 1976; Egeth and Sager, 1977; Colavita and Weisberg, 1979). In the classical paradigm of the Colavita effect, visual, auditory, and audiovisual stimuli are randomly presented, and two response keys are pre-specified to the visual (“visual key”) and the auditory (“auditory key”) targets, respectively. Participants are instructed to press the “visual key” whenever the visual target is presented, press the “auditory key” whenever the auditory target is presented, and make both responses whenever the visual and the auditory targets are simultaneously presented (i.e., the bimodal audiovisual stimuli). Although participants are able to respond rapidly and accurately to the unimodal stimuli, they often fail in responding to the auditory component of the bimodal audiovisual stimuli and respond almost exclusively to the visual component (Koppen and Spence, 2007a,b,c,d; Koppen et al., 2008, 2009; Sinnett et al., 2007; Spence, 2009; Spence et al., 2012).

The classical studies of visual dominance effect mainly focus on the proportions of incorrectly responded bimodal trials, i.e., the bimodal trials in which only the “visual key” (Visual_Only) or only the “auditory key” (Auditory_Only) is pressed. Because the number of Auditory_Only trials is so limited (less than 10% of the bimodal trials) (e.g., Colavita, 1974, 1982; Colavita et al., 1976; Egeth and Sager, 1977; Colavita and Weisberg, 1979) such that not enough trials can be collected to meet the least trial number required by ERP and fMRI studies, therefore, the neural correlates of the visual dominance effect remained unknown for a long time. Recently, two studies adopted a new way of data analysis to solve this problem (Huang et al., 2015; Yue et al., 2015). In addition to the classical analysis focusing on the incorrect trials as Colavita (1974), we further analyzed the proportions of the correct bimodal trials, i.e., the bimodal trials in which participants made responses both to the visual and the auditory components. Specifically, in most of these correct bimodal trials, although participants made both responses, they could not press the visual and auditory keys strictly simultaneously: either the visual response preceded the auditory response or vice versa. Results from the two studies consistently showed that the ratio of “vision preceded audition” (Visual_Auditory) bimodal trials was significantly larger than the ratio of “audition preceded vision” (Auditory_Visual) bimodal trials, indicating vision’s dominance over audition. Moreover, even when visual responses were preceded by auditory responses, they recovered more quickly from previous responses, further demonstrating the visual dominance effect. We thus found a novel way of defining the visual dominance effect, and more importantly, we now have enough trial numbers to calculate the neural correlates underlying the visual dominance effect by post hoc categorizing bimodal trials into the Visual_Auditory condition and the Auditory_Visual trials.

In a previously published study we used ERP and fMRI techniques to investigate the neural causes of visual dominance effect (Huang et al., 2015). ERP results indicated that the visual dominance effect was associated with a stronger post-perceptual positivity: the ERPs of the Visual_Auditory and Auditory_Visual conditions kept overlapping until 250 ms after stimulus onset, and the Visual_Auditory ERP turned significantly more positive than the Auditory_Visual ERP at about 250 to 400 ms after stimulus onset, with the strongest difference over the centroparietal regions. In addition, the fMRI results contrasting the Visual_Auditory and Auditory_Visual conditions showed that, when vision dominated audition, the dorsal visual stream showed not only increased activity, but also enhanced functional connectivity with the sensorimotor cortex and inferior frontal cortex. These results together indicated that the outcome of multisensory competition depended on the dynamic interaction between sensory system and the fronto-sensorimotor system, and the visual dominance effect implicated the prioritizing of visual information into the motor system.

In order to examine the neurophysiological mechanism of how visual information is prioritized into the motor system when vision dominates audition, the present study reanalyzes the EEG data from the previously published study (Huang et al., 2015) focusing on a particular ERP component, the lateralized readiness potential (LRP). The LRP is proposed to be a neural marker of motor activation (Smid et al., 1987; Coles et al., 1988; de Jong et al., 1988; Gratton et al., 1988). When preparing a motor response, the potential over the motor cortex contralateral to the responding hand develops more negatively relative to that over the ipsilateral motor cortex. So that, by subtracting the activity recorded over the ipsilateral cortex from the activity over the contralateral cortex, the LRP is revealed, which indexes response selection and preparation (Smulders and Miller, 2012). In the present study, we examine the LRP time course profiles of the Visual_Auditory and the Auditory_Visual bimodal trials to reveal how information from different sensory modalities is transported to the motor system, activates motor response, and eventually determines the outcome of multisensory competition. Moreover, two types of LRPs were calculated in the present study, the stimulus-locked LRP (S-LRP) and the response-locked LRP (R-LRP), which would help with further differentiating the processes of response preparation and execution. Generally, it has been considered that the time between the stimulus encoding and the S-LRP onset reflected response preparation process, while the time between the R-LRP onset and the behavioral response reflected response execution process (Masaki et al., 2004; Schröter and Leuthold, 2009; Xu et al., 2015; Miller, 2016). By examining both types of LRPs, the present study unraveled at which stage of motor process the Colavita visual dominance effect emerged.

## Materials and Methods

The current study is based on a reanalysis of EEG data from a previously published study (Huang et al., 2015). In the following, we focus on the procedures unique to the present reanalysis of those data.

### Participants

Twenty (11 females, 18–26 years old) healthy adult participants volunteered to take part in the present study. The participants were all right-handed, with normal hearing and normal or corrected-to-normal visual acuity. None of them had a history of neurological or psychiatric disorders. All the participants gave their informed consent before the study in accordance with the Declaration of Helsinki. This study was approved by the Ethics Committee of the School of Psychology, South China Normal University.

### Stimuli and Experimental Design

The auditory target was a 4000 Hz pure tone with the length of 50 ms, and the visual target was a white sphere with a radius of 1.5° visual angle and a luminance of 1.9 cd/m^2^. The default visual display was a white cross that measured 1° × 1° of visual angle on a gray background (red–green–blue value, 128, 128, and 128). Stimulus presentation was controlled with Presentation Software package (Neurobehavioral Systems).

There were three types of trials: (1) unimodal auditory trials in which only the auditory target was presented for 50 ms (i.e., the Auditory_Single condition); (2) unimodal visual trials in which only the visual target was presented for 50 ms at the center of the screen (i.e., the Visual_Single condition); and (3) bimodal trials in which the auditory and the visual targets were presented simultaneously for 50 ms. The three types of trials were presented randomly. In the experiment, participants were instructed to press one button on the response pad with the thumb of one hand if the auditory target appeared, press the other button with the thumb of the other hand if the visual target appeared, and press both buttons as simultaneously as possible if both the auditory and the visual targets appeared. The mapping between the two response buttons and the visual and auditory targets was counterbalanced across participants. Participants were told to press down the two buttons as simultaneously as possible in the bimodal trials via strict instructions before the formal experiments: (1) participants were informed explicitly of the existence of the bimodal trials and (2) were instructed to press the visual key and the auditory key as simultaneously as possible in the bimodal trials.

Most critically, the bimodal trials were *post hoc* categorized into the following six types of behavioral conditions based on participants’ online performance: (1) the Visual_Auditory (VA) responses, in which participants first responded to the visual component and then to the auditory component; (2) the Auditory_Visual (AV) responses, in which participants first responded to the auditory component and then to the visual component; (3) the “Simultaneous” responses, in which participants responded simultaneously to the auditory and the visual components by pressing down the two response buttons at the same time; based on the uncertainty errors (2–5 ms) recorded by the Presentation software, the bimodal trials, in which the absolute RT difference between the responses to the visual and the auditory components was <5 ms (| Visual_RT > Auditory_RT| < 5 ms), were categorized as the simultaneous trials as well; (4) the Visual_Only responses, in which participants responded only to the visual component but not to the auditory component; (5) the Auditory_Only responses, in which participants responded only to the auditory component but not to the visual component; and (6) the “Missed” trials, in which no responses were recorded.

### Procedure

The experiment was conducted in a dimly lit and soundproof room. The visual target was presented on an LCD monitor. The auditory target was delivered via a loudspeaker that was positioned directly behind the LCD monitor to ensure that the auditory tone sounded like it was coming from the same central spatial position as the visual target. Participants were instructed to fixate at the central fixation cross throughout the experiment without moving their eyes and to detect the appearance of the target stimuli by pressing the pre-specified buttons. The mapping between the two response buttons and the auditory and visual targets was counterbalanced across participants.

The experiment consisted of 10 blocks in total, and each block included 80 Auditory_Single trials, 80 Visual_Single trials, and 40 bimodal trials, which were mixed randomly. Each trial was followed by a time interval that was selected randomly among 1350, 1450, 1550, 1650, and 1750 ms. The temporal order of trials was randomized for every participant. Before the experiment, all participants were familiarized with the tasks and the experimental setup by a training session of 10 min.

### ERP Recording and Analysis

EEGs were continuously recorded from 64 Ag/AgCl electrodes (10–20 System) with BrainAmp DC amplifiers (low-pass = 100 Hz, high-pass = 0.01 Hz, sampling frequency = 500 Hz). Signals were referenced online to the left mastoid and re-referenced offline to the two mastoids average. Electrooculograms (EOGs) were recorded using three facial bipolar electrodes with two being placed on the outer canthi of each eye to record the horizontal EOG and one being positioned in the inferior areas of the left orbit to record the vertical EOG. All the electrode impedances were kept below 5 kΩ.

### Analysis of Behavioral Data

For the behavioral data, the outlier trials, in which the RTs exceeded 3 standard deviations (SDs) in each condition, were excluded from analysis (0.9% of the overall data were excluded as outliers). Based on the online responses in the bimodal trials, we differentiated between the six behavioral conditions in the bimodal trials. The ratio of each behavioral condition was calculated as the proportion between the number of bimodal trials in each behavioral condition and the overall number of bimodal trials. For RTs, we focused our analysis on RTs to the visual and the auditory components in the two critical behavioral conditions in the bimodal trials, i.e., the Visual_Auditory and the Auditory_Visual trials. Omissions, incorrect responses, and outlier trials for each condition were first excluded from additional analysis. Mean RTs of the remaining trials were then calculated for each condition and submitted to a 2 (Type of Response: responses to the visual components vs responses to the auditory components) × 2 (Response Order: the first response vs the second response) repeated-measures ANOVA.

### Analysis of LRP

During offline data analysis, we classified post hoc the bimodal trials into six types based on the participants’ online responses, and segmented the EEGs of the bimodal trials according to the types of bimodal behavioral conditions and of the two types of unimodal trials. S-LRP epochs ranged from –200 to 800 ms time-locking at stimulus presentation and were corrected to 200 ms pre-stimulus baseline. R-LRP epochs ranged from –800 to 200 ms time-locking at the first response and were corrected to a baseline between –800 to –600 ms before response. Segments with EEG exceeding ±100 μV relative to baseline and with EOG exceeding ±80 μV relative to baseline were excluded. For each participant and each condition of interest (i.e., the Visual_Single, Auditory_Single, Visual_Auditory, and Auditory_Visual conditions), the resulting trial number for averaging was above 45, except for two participants, who were discarded from following analysis. In order to balance the trial numbers for averaging across the four conditions, for each participant, a 1,000-time boot-strapping procedure was used to randomly select a same number of trials from each condition.

The LRPs were computed from ERPs recorded from C3/C4 sites. For each response, the ipsilateral ERPs were subtracted from the contralateral ERPs relative to the responding hand. The difference waveforms were averaged across responding hands separately for each condition and participant. Potential confounds from non-motor lateralization could be excluded or minimized because the mapping between the responding hands and the visual and auditory targets was counter-balanced across participants and all the stimuli were centrally presented. LRP onsets were measured by the jackknife-based procedure (Miller et al., 1998). An absolute criterion (–0.5 μV) was used because the amplitude of LRP waveforms differed between conditions (Miller et al., 1998). Each averaged LRP was low-pass filtered offline at 15 Hz before onset measurement to reduce high-frequency noise. The LRP onset was determined as the time point when the LRP amplitude exceeded –0.5 μV. *F* and *t* values were corrected when applying the jackknife-based *t*-tests and ANOVAs: *t*-values were corrected as *t*_C_ = *t*/(n-1), and *F* values were corrected as *F*_C_ = *F*/(n-1)^2^ (Miller et al., 1998; Ulrich and Miller, 2001).

### Results

The behavioral results have been reported in the previously publication, thus, we reported here only those necessary to demonstrate the visual dominance effect (for more detailed analysis, see Huang et al., 2015). Most importantly, we focused on the EEG data reanalysis, i.e., the LRP results.

### Behavioral Data

For unimodal trials, RTs did not differ between the Auditory_Single (462 ms) and the Visual_Single trials (462 ms), *t*(17) = 0.03, *p* = 0.974, *d* < 0.01, supporting that the simple detection for single auditory stimulus or for single visual stimulus were comparable.

**FIGURE 1:**
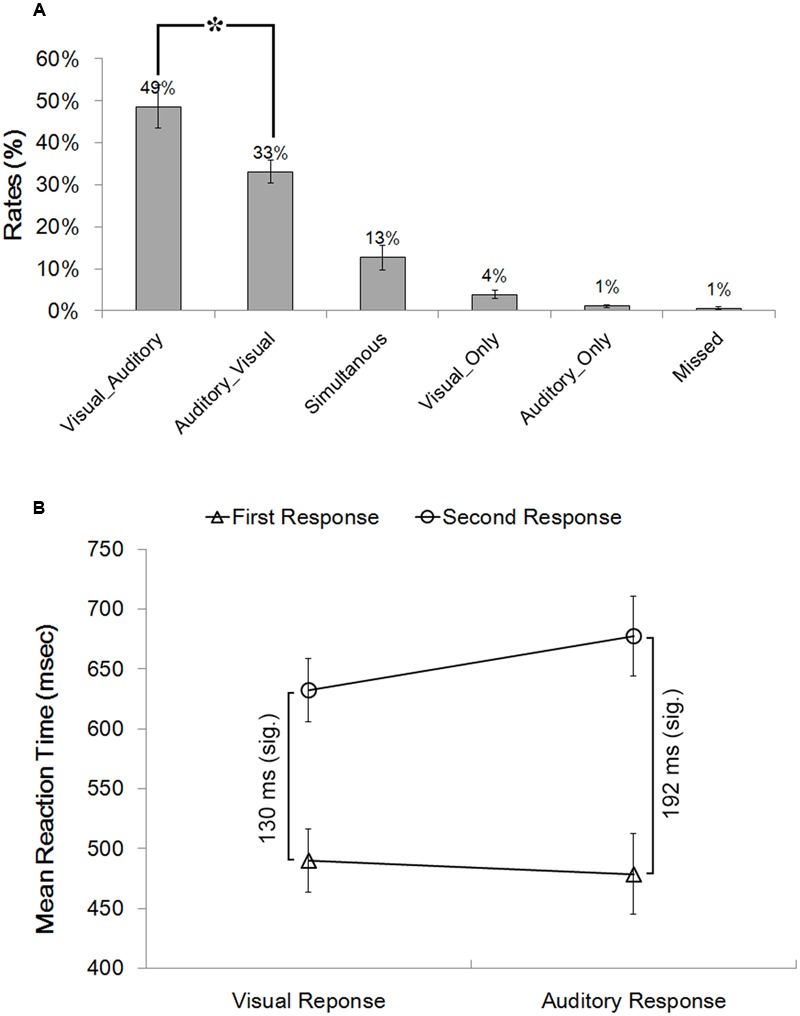
**Behavioral results. (A)** Proportions of the six different types of behavioral conditions in the bimodal trials. Bimodal trials were categorized into six behavioral conditions based on participants’ online performance: (1) the Visual_Auditory trials in which participants first responded to the visual component and then to the auditory component; (2) the Auditory_Visual trials in which participants first responded to the auditory component and then to the visual component; (3) the “Simultaneous” trials in which participants responded simultaneously to the auditory and the visual components; (4) the Visual_Only trials in which participants responded only to the visual component; (5) the Auditory_Only trials in which participants responded only to the auditory component; and (6) the “Missed” trials in which no responses were made. **(B)** Reaction times to the visual and the auditory components of the Visual_Auditory and the Auditory_Visual trials shown as a function of response order. ^∗^*p* < 0.05. The error bars indicate standard errors.

Bimodal trials were post hoc categorized into six types of behavioral conditions based on participants’ online performance. The most critical types were the Visual_Auditory and Auditory_Visual trials. In these two types of trials, visual responses preceded auditory responses, or vice versa, which revealed the dominance effect of one particular modality over the other one. The proportion of the VA responses (49%) was significantly higher than the proportion of the AV responses (33%) (**Figure 1A**), *t*(17) = 2.07, *p* = 0.054, *d* = 0.49, which revealed a significant visual dominance effect that when facing bimodal audiovisual information, visual detection responses preceded auditory responses more frequently than vice versa. In addition, the classical Colavita effect was also found: the proportion of Visual_Only response (4%) was significantly higher than the proportion of the Auditory_Only response (1%) (**Figure 1A**), *t*(17) = 3.00, *p* = 0.008, *d* = 0.71.

RTs in the VA and AV bimodal trials were submitted to a 2 (Type of Response: auditory response vs. visual response) × 2 (Response Order: first vs. second) repeated-measures ANOVA (**Figure 1B**). The main effect of the Type of Response was not significant, *F*(1,17) = 2.55, *p* = 0.128, η^2^ = 0.131. The main effect of the Response Order was significant, *F*(1,17) = 70.44, *p* < 0.001, η^2^ = 0.806, indicating that the first responses (501 ms) were significantly faster than the second responses (662 ms). Most critically, the interaction between the Type of Response and the Response Order was significant, *F*(1,17) = 17.86, *p* = 0.001, η^2^ = 0.512. Planned paired *t* tests revealed that for the second responses, RTs to the visual components in AV trials (640 ms) were significantly faster than RTs to auditory components in VA trials (685 ms), *t*(17) = 3.90, *p* = 0.001, *d* = 0.92. By contrast, for the first responses, no significant difference was found between the RTs to the visual (510 ms) and the auditory (493 ms) first responses, *t*(17) = 1.41, *p* = 0.178, *d* = 0.33. Moreover, the extent of response delay in responding to the visual component in the Auditory_Visual trials as compared to the Visual_Auditory trials (130 ms) was significantly smaller than the extent of response delay in responding to the auditory component in the Visual_Auditory trials relative to the Auditory_Visual trials (192 ms), *t*(17) = 4.20, *p* = 0.001, *d* = 0.92.

### LRP Data

**FIGURE 2:**
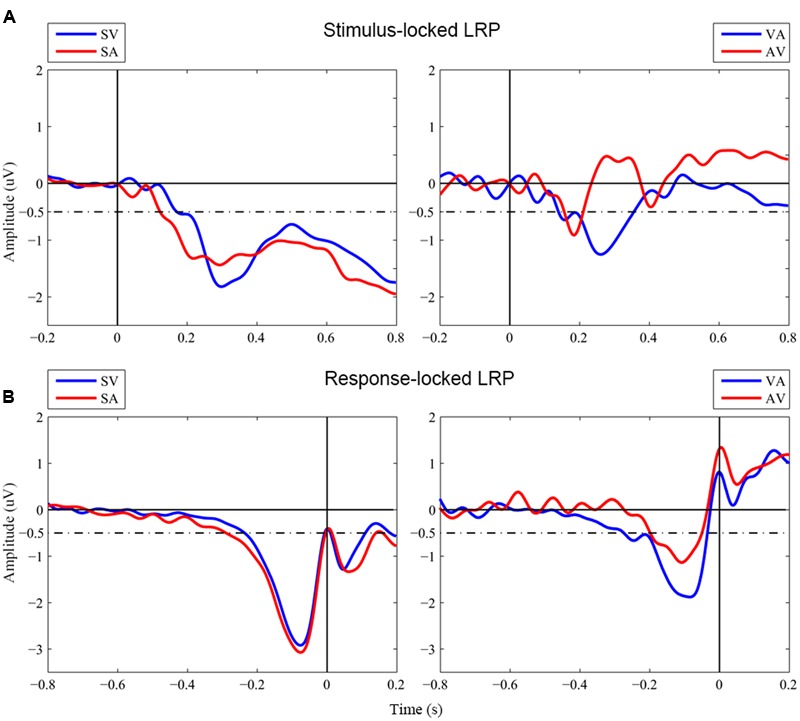
**Grand-averaged waveforms of LRPs. (A)** Stimulus-locked LRP waveforms. Stimulus onset is at time zero. **(B)** Response-locked LRP waveforms. Response onset is at time zero. SV, Visual_Single; SA, Auditory_Single; VA, Visual_Auditory; AV, Auditory_Visual. Dashed line indicated the –0.5 μV criterion used to measure the LRP onsets in the jackknife-based procedure.

**Figure 2** shows grand-averaged LRP waveforms across the four conditions of interest, i.e., two unimodal conditions, Visual_Single and Auditory_Single, and two bimodal conditions, Visual_Auditory and Auditory_Visual. Onset latencies of S-LRP and R-LRP measured with the jackknife procedure are depicted in **Table 1**. Two (Component Number: unimodal vs. bimodal) by two (Component Modality: visual vs. auditory) repeated-measures ANOVAs were performed on the onset latencies of S-LRP and R-LRP, respectively.

**Table 1:** Mean LRP onsets (ms) estimated by the jackknifing method.

	S-LRP onsets (SEM)	R-LRP onsets (SEM)
Visual_Single	176 (4)	–236 (3)
Auditory_Single	124 (1)	–288 (3)
Visual_Auditory	148 (4)	–275 (4)
Auditory_Visual	158 (2)	–197 (2)

S-LRP onsets showed no significant main effect neither for Component Number, *F*_C_(1,17) < 1, *p* = 0.973, nor for Component Modality, *F*_C_(1,17) < 1, *p* = 0.794. The interaction was not significant either, *F*_C_(1,17) < 1, *p* = 0.343. In contrast, R-LRP onsets showed a significant interaction between Component Number and Component Modality, *F*_C_(1,17) = 5.13, *p* = 0.037. Specifically, the R-LRP to a visual target emerged 39 ms earlier when it was accompanied by an auditory target (i.e., the Visual_Auditory trial, -275 ms before response) than when it was presented alone (i.e., the Visual_Single trial, –236 ms before response), albeit not significantly, *t*_C_(17) < 1, *p* = 0.368; while, with an opposite pattern, the R-LRP to an auditory target was 91 ms delayed when it was accompanied by an visual target (i.e., the Auditory_Visual trial, –196 ms before response) than when it was presented alone (i.e., the Auditory_Single trial, -288 ms before response), *t*_C_(17) = 1.94, *p* = 0.069.

## Discussion

In order to investigate the neurophysiological correlates of the visual dominance effect, the present study examined the time course of the LRP during the processing of multisensory competition. Behavioral data indicated the dominance of vision over audition: Participants failed to respond to the auditory component of bimodal targets significantly more often than to the visual component, i.e., the classical Colavita effect (Colavita, 1974; Egeth and Sager, 1977; Spence, 2009; Spence et al., 2012). In addition, the visual dominance effect was further strengthened by the higher frequency with which the visual responses preceded the auditory responses than vice versa. Moreover, even when visual responses were preceded by auditory responses, they recovered more quickly from the previous responses (Huang et al., 2015; Yue et al., 2015).

More importantly, the results of LRP indicated that, it was the relative timing of R-LRP between the unimodal and bimodal trials that was related to the nature of visual dominance. When the visual and auditory stimuli were presented simultaneously and competed for responses, the relative delays of R-LRP between the unimodal and bimodal trials were asymmetric for visual and auditory components. Specifically, in terms of the comparison between the unimodal and the bimodal trials, the onset of R-LRP to the auditory components of the Auditory_Visual trials (–196 ms before response, **Figure 2B** red line in the right panel) was significantly delayed about 91 ms compared to the onset of the LRP to the unimodal auditory targets (-288 ms before response, **Figure 2B** red line in the left panel), whereas, the onset of R-LRP to the visual components of the Visual_Auditory trials (–275 ms, **Figure 2B** blue line in the right panel) was comparable to, or even earlier than, the R-LRP to the unimodal visual targets (–236 ms, **Figure 2B** blue line in the left panel). Thus, the onset of R-LRP to the auditory target was delayed, when it was paired with a simultaneous visual target in the bimodal trials, compared to the unimodal auditory trials when auditory information was not paired with visual information. For the visual information, however, the onset of R-LRP was comparable irrespective of whether the visual information was paired with auditory information or not.

In contrast, although the waveforms of S-LRPs appeared to differ on the onset latency or shape, we did not observe any statistically significant difference of S-LRPs across conditions. The onset of S-LRP was considered to represent the duration of process preceding hand-specific preparation (i.e., perceptual processing and response selection); while the onset of R-LRP was used to infer the duration of response-related process (Masaki et al., 2004; Schröter and Leuthold, 2009; Xu et al., 2015; Miller, 2016). Therefore, the present LRP findings implicated the involvement of the Colavita visual dominance effect at the motor response execution stage. Consistently, ERP investigation of the Colavita effect found no significant difference in the early perceptual process between the ERPs of Visual_Auditory and Auditory_Visual trials, which suggested that the Colavita effect took place at the post-perceptual phase and emphasized the prioritizing access of visual information in the motor system (Huang et al., 2015).

Previous studies have documented that the Colavita effect is robust irrespective of a variety of experimental manipulations (e.g., of stimulus intensity, stimulus type, stimulus position, response demands, attention, arousal) (Colavita, 1974, 1982; Colavita et al., 1976; Egeth and Sager, 1977; Koppen et al., 2008, 2009; Koppen and Spence, 2007c,d; Sinnett et al., 2007, 2008; Spence et al., 2012). Attempts to explain the Colavita visual dominance effect came from studies looking at the effect of accessory stimuli presented in one modality on the speeded responds to targets presented in another modality (Sinnett et al., 2008), and from studies looking at the sensitivity and criterion of participants’ responses in the Colavita task (Koppen et al., 2009). Sinnett et al. (2008) found that, in a speeded detection task, responses to auditory targets were significantly slowed down when they were accompanied by an accessory visual stimulus than when they were presented alone. Whereas, an auditory accessory stimulus speeded up the responses to the visual targets. Further, by using signal detection theory, Koppen et al. (2009) found a significant decrease in participants’ sensitivity (*d′*) to the auditory stimulus when presented concurrently with an accessory visual stimulus, whereas an accessory auditory stimulus had no significant effect on visual sensitivity. In addition, response criterion (*c*) was significantly lower in the bimodal than in the unimodal conditions for both the visual and the auditory stimuli, with the criterion decrease of a larger magnitude for the visual than for the auditory stimulus. Taken recent evidence together, it was proposed that the most plausible model of the Colavita visual dominance effect would appear to be one combining an asymmetric lowering of the criterion for responding to auditory and visual targets, with an asymmetric cross-modal effect on the rate of information accrual (Spence et al., 2012). In line with this proposal, the present study revealed the LRP indexes of these asymmetric cross-modal effects: The onset of R-LRP to an auditory target was delayed by a simultaneously presented visual stimulus, compared to when it was presented by itself. However, the R-LRP to visual targets was not affected by an auditory accessory stimulus. The LRP results of the present study therefore indicated a prepotency of visual modality in activating response preparation in the motor system. Consistently, the recent fMRI study on the Colavita visual dominance effect found that the dorsal visual stream was activated during visual dominance and showed increased functional connectivity with sensorimotor cortex and inferior frontal cortex (Huang et al., 2015), which might reflect the underpinning neural substrate to prioritize the flow of visual information into the motor system.

Highly relevant to the present study, a bunch of research into a phenomenon called warning effect found that an accessory auditory stimulus paired with a visual stimulus affected the S-LRP but not the R-LRP (Hackley and Valle-Inclan, 1998, 1999). In the warning effect paradigm, the accessory stimulus was presented prior to the target (e.g., 30 ms). More importantly, the accessory stimulus was task-irrelevant so that it served as a warning signal. As a consequence, behaviorally, the task-irrelevant accessory stimulus shortened the RT to the target (Hackley, 2009). In contrast, in the Colavita paradigm, both auditory and visual stimuli were task-relevant targets competing for response. Accordingly, the accessory auditory stimulus in the Colavita paradigm lengthened the RT to the visual target. Consistent with the behavioral results, the neurophysiological results differed between the two paradigms: in the warning effect paradigm, the task-irrelevant accessory stimulus advanced the onset of S-LRP; while in the Colavita paradigm, the task-relevant completing stimulus delayed the onset of R-LRP. The existing results together indicated that the task-relevance of the concurrent stimulus determined the nature of its impact on the motor system.

## Conclusion

Aimed at investigating the neurophysiological correlates of the Colavita visual dominance effect, the present study found that, when vision dominated audition in multisensory competition, the R-LRP to the auditory target was significantly delayed by a simultaneously presented visual target, whereas the R-LRP to visual target did not affected by the concurrent auditory target, thus suggesting a prioritizing role of the visual information in motor activation.

## Ethics Statement

All the participants gave their informed consent before the study in accordance with the Declaration of Helsinki. This study was approved by the Ethics Committee of the School of Psychology, South China Normal University.

## Author Contributions

QC designed research; YL, ML, SH, and BZ performed research; YL, ML, SH, BZ, and QC analyzed data; WZ, XL, and QC wrote the paper.

## Conflict of Interest Statement

The authors declare that the research was conducted in the absence of any commercial or financial relationships that could be construed as a potential conflict of interest.
